# Decoration of Nanovesicles with pH (Low) Insertion Peptide (pHLIP) for Targeted Delivery

**DOI:** 10.1186/s11671-018-2807-8

**Published:** 2018-12-04

**Authors:** Federica Rinaldi, Patrizia N. Hanieh, Elena Del Favero, Valeria Rondelli, Paola Brocca, Mohan C. Pereira, Oleg A. Andreev, Yana K. Reshetnyak, Carlotta Marianecci, Maria Carafa

**Affiliations:** 1 Center for Life Nano Science@Sapienza, Fondazione Istituto Italiano di Tecnologia, Viale Regina Elena, 291, 00161 Rome, Italy; 2 Department of Drug Chemistry and Technology, University of Rome “Sapienza”, P.le A. Moro, 5, 00185 Rome, Italy; 3 Department of Medical Biotechnologies and Translational Medicine, University of Milan, LITA, Via F.lli Cervi 93, 20090 Segrate, Italy; 4 Physics Department, University of Rhode Island, 2 Lippitt Rd, Kingston, RI 02811 USA

**Keywords:** pHLIP, Liposomes, Niosomes, pH-sensitivity, Cryo-TEM, SAXS

## Abstract

Acidity at surface of cancer cells is a hallmark of tumor microenvironments, which does not depend on tumor perfusion, thus it may serve as a general biomarker for targeting tumor cells. We used the pH (low) insertion peptide (pHLIP) for decoration of liposomes and niosomes. pHLIP senses pH at the surface of cancer cells and inserts into the membrane of targeted cells, and brings nanomaterial to close proximity of cellular membrane. DMPC liposomes and Tween 20 or Span 20 niosomes with and without pHLIP in their coating were fully characterized in order to obtain fundamental understanding on nanocarrier features and facilitate the rational design of acidity sensitive nanovectors. The samples stability over time and in presence of serum was demonstrated. The size, ζ-potential, and morphology of nanovectors, as well as their ability to entrap a hydrophilic probe and modulate its release were investigated. pHLIP decorated vesicles could be useful to obtain a prolonged (modified) release of biological active substances for targeting tumors and other acidic diseased tissues.

**Electronic supplementary material:**

The online version of this article (10.1186/s11671-018-2807-8) contains supplementary material, which is available to authorized users.

## Introduction

About a century ago, Paul Ehrlich floated the idea of realizing “magic bullets” to specific and more effective delivery of drugs [1]. The “magic bullets” should be able to protect the delivered drug from a harsh environment, decrease the side effects by targeting drug to diseased tissue, improving drug pharmacokinetic and pharmacodynamic, and modulating drug release [2].

In 1965, Bangham observed phospholipid-based vesicles for the first time [3] and, in the following years, Gregory Gregoriadis established the concept that liposomes could encapsulate drugs and then be used as drug delivery systems. In particular, they are composed by phospholipid closed bilayers (lamellae), where the hydrophobic lipid chains are closed between two hydrophilic headgroups layers. The closed bilayer surrounds an aqueous core, thus allowing for the localization of lipophilic or hydrophilic drugs respectively [4, 5].

Liposome size is a critical parameter in influencing the carrier fate after administration, in terms of plasma proteins absorption, recognition by reticuloendothelial system (RES), circulation half time, and cellular trafficking. Nanosized carriers can favor cell internalization and tumor targeting, thus many researchers have been focusing on “nanonization” [6–9].

In order to obtain more versatile and more economic nanocarriers, synthetic surfactants have been employed to obtain liposome-like drug delivery systems. Non-ionic surfactants are widely used at this purpose, and are able to self-assemble into unilamellar or multilamellar vesicles (*non*-*ionic liposomes*, niosomes, or non-ionic surfactant vesicles). Sorbitan esters surfactants (Spans®) are lipophilic substances widely used in niosome preparation. In order to prolong the vesicles circulation time and obtain “stealth” nanocarriers, polyethylene glycol (PEG) incorporation is a gold standard approach: through this conjugation, ethoxyethylatedsorbitan esters surfactants (Tweens®) are obtained. Both Span and Tween are characterized by a different hydrophilic/lipophilic balance (HLB) value and the choice of the surfactant allows preparing niosomes with the desired properties [10]. Furthermore, the addition of cholesterol is used for the enhancement of the bilayer stability by stretching out the surfactant tails, affecting surfactant’s gel to liquid phase transition temperature and conferring rigidity of the lipophilic bilayer [11, 12].The “optimized” nanocarrier is designed to improve formulation and/or enhance targeting [10].

Nowadays, cancer is one of the main causes of death in the world. Current therapeutic approaches have a number of limitations including non-efficient drug delivery to tumors and lack of tumor targeting associated with undesirable and dangerous side effects, which nanotechnology approaches might help to overcome [13].

Currently, various targeting approaches are under development. Most of them are based on the targeting of particular biomarkers overexpressed on the surface of cancer cells. However, due to the fact that human tumors are very heterogeneous, more general approaches of tumor targeting will be much more advantageous. Acidity at the surface of cancer cells is a hallmark of tumor microenvironments, and it does not depend on tumor perfusion, thus it may serve as a general biomarker for targeting tumor cells [14]. pH (low) insertion peptide (pHLIP) technology is rapidly developing for targeting imaging and therapeutic small molecules, as well as nanomaterials to tumors. pHLIP senses pH at the surface of cancer cells and inserts into the membrane of targeted cells [15, 16]. The insertion mechanism of pHLIP is triggered by the protonation of negatively charged residues of the peptide at low pH (pH < 7.0). This leads to an increase of peptide’s hydrophobicity thus shifting the equilibrium toward partitioning of the peptide into the bilayer [17]. Nanocarriers decorated with pHLIPs are biocompatible, can target tumors, and demonstrate enhanced cellular uptake by cancer cells. Among investigated pHLIP-coated nanoparticles are lipid, polymer, and metal-based nanomaterials [18–21].

The aim of the present work is to fully characterize novel vesicular nanocarriers decorated by pHLIP in order to obtain fundamental understanding on nanocarrier features and facilitate the rational design of acidity sensitive nanovectors.

## Materials and Methods

### Materials

Polyoxyethylenesorbitan monolaurate (Tween 20), sorbitan monolaurate (Span 20), cholesterol (Chol), Hepes salt {*N*-(2-idroxyethyl) piperazine-*N***′**-(2-ethanesulfonic acid)}, human serum, Sephadex G-75, calcein, and diphenylhexatriene (DPH) were purchased from Sigma-Aldrich. 1,2-Dimyristoyl-*sn*-glycero-3-phosphocholine (DMPC) and 1,2-dioleoyl-sn-glycero-3-phosphoethanolamine-*N*-[4-(p-maleimidophenyl)butyramide] sodium salt (DSPE-maleimide) were purchased from Avanti Polar Lipids and pyrene was obtained from Fluka. pHLIP peptide (ACEQNPIYWARYADWLFTTPLLLLDLALLVDADEGT) was synthesized and purified by CS Bio. All other products and reagents were of analytical grade.

### Synthesis of DSPE-pHLIP

pHLIP was conjugated with DSPE lipids in methanol by the covalent conjugation of DSPE-maleimide with the single cysteine residue at the N-terminus of pHLIP, as it was described previously [18, 21, 22]. Briefly, 5 mg of peptide dissolved in 250 μL methanol (blown with argon) and DSPE-maleimide (from 9.9 mM stock solution) dissolved in chloroform were mixed at a molar ratio of 1:1. Reaction mixture was kept at room temperature for about 2–6 h until the conjugation was completed. The reaction progress in conjugation of DSPE-malemide with pHLIP was monitored by the RP-HPLC using a gradient from 25 to 80% acetonitrile in water containing 0.05% TFA by monitoring a decrease of peak corresponding to the unlabeled pHLIP in the reaction mixture. The synthesized construct was characterized by SELDI-TOF mass-spectrometry. The concentration of DSPE-pHLIP conjugate was determined by absorbance using the molar extinction coefficient for pHLIP: *ε*_280_ = 13,940 M^−1^ cm^−1^.

### Vesicles Preparation and Purification

The thin layer evaporation method was used to prepare non-ionic surfactant (from Tween 20 or Span 20) and phospholipid (from DMPC) vesicles, with and without pHLIP. In each vesicle formulation, cholesterol was added in different molar ratios (Table 1) [23].

**Table 1:** Sample composition

Sample	Tween 20 (mM)	Span 20 (mM)	DMPC (mM)	Chol (mM)	DSPE-pHLIP (mM)
NioTween20	15.0	–	–	15.0	–
NioSpan20	–	15.0	–	15.0	–
LipoDMPC	–	–	49.4	29.7	–
NioTween20-pHLIP	15.0	–	–	15.0	0.1
NioSpan20-pHLIP	–	15.0	–	15.0	0.1
LipoDMPC-pHLIP	–	–	49.4	29.7	0.1

Sample composition has been optimized choosing previously well-characterized structures [24, 25] at which the same amount of pHLIP has been added.

The lipophilic components were first dissolved in CHCl_3_:CH_3_OH mixture (3:1); the organic solvent was then removed under vacuum at different temperatures depending on the sample. The obtained film was hydrated with 5 mL of Hepes buffer (0.01 M pH 7.4) or sodium calcein solution 10^−2^ M. The suspension was vortex-mixed for about 5 min, followed by sonication (see Additional file 1: Table S1, supporting information) using a microprobe operating at 20 kHz (VibraCell-VCX 400-Sonics, Taunton, MA, USA). LipoDMPC sonication was conducted under inert atmosphere to prevent oxidation.

The vesicle suspension was then purified by gel permeation chromatography using Sephadex G-75 (glass column of 50 × 1.2 cm) and Hepes buffer as eluent. Afterwards, the purified vesicle suspension was filtered by means of cellulose filters with the appropriate pore diameter.

The same preparation method was used to prepare pHLIP-coated niosomes and liposomes.

### Dynamic Light Scattering Measurements

The average size and size distribution of the vesicles were measured at *T* = 25 °C by dynamic light scattering (DLS), using a Malvern NanoZetaSizer ZS90, equipped with a 5 mW HeNe laser (wavelength λ = 632.8 nm) and a digital correlator. The normalized autocorrelation functions of the scattered intensity at 90° angle were analyzed by the Contin algorithm to obtain the distribution of the particles diffusion coefficient *D*, hence the distribution of the effective hydrodynamic radius *R*_H_ of the vesicles via the Stokes-Einstein relation *R*_H_ = *K*_B_*T*/6πη*D*, where *K*_B_*T* is the thermal energy and η is the solvent viscosity. The width of the size distribution of niosomes/liposomes is rather small but non-negligible. The values reported in Table 2 correspond to the intensity-weighted average hydrodynamic diameter of the particles [26].

**Table 2:** Samples characterization in hydrodynamic diameter, ζ-potential, and PDI

Sample	Hydrodynamic diameter ± SD	ζ-Potential (mV) ± SD	PDI
NioTween20	162.0 ± 2.0	− 22.8 ± 0.2	0.26
NioTween20-pHLIP	168.5 ± 2.3	− 19.5 ± 0.6	0.37
NioSpan20	165.5 ± 3.3	− 36.4 ± 0.9	0.35
NioSpan20-pHLIP	156.3 ± 1.0	− 38.4 ± 2.3	0.21
LipoDMPC	139.7 ± 2.4	− 15.9 ± 0.4	0.15
LipoDMPC-pHLIP	129.8 ± 2.0	− 10.8 ± 0.4	0.13

### ζ-Potential Measurements

The electrophoretic mobility measurements were carried out by the laser Doppler electrophoresis technique, using the Malvern NanoZetaSizer ZS90 apparatus. The mobility *u* was converted into the *ζ*-potential using the Smoluchowski relation ζ = uη/є, where η and є are the viscosity and the permittivity of the solvent phase, respectively [27].

### Small Angle X-Ray Scattering

Small angle X-ray scattering (SAXS) experiments were carried out at European Synchrotron Radiation Facility (ESRF, Grenoble, France). The use of the ID02 high-brilliance beamline allowed to perform measures on dilute solutions in the region of momentum transfer 0.1 nm^−1^ ≤ *q* ≤ 6 nm^−1^, *q* = (4π/λ)sin(θ/2), where θ is the scattering angle and λ = 0.1 nm the X-ray wavelength. The corresponding investigated length-scale is between 1 and 60 nm, suitable for accessing the internal structure of niosomal and liposomal vesicles. All experiments were performed at *T* = 25 °C with short irradiation time, 0.1 s, to prevent radiation damage. For each sample, the scattered intensity at different scattering angles was captured on a 2D detector, then angularly regrouped, subtracted for the background and solvent contributions, and analyzed to obtain information on the shape and internal structure of the vesicles in solution.

In the case of unilamellar vesicles, the closed bilayer was modeled with three concentric shells corresponding to the external headgroups, the hydrophobic chains, and the internal headgroups. A Schulz distribution for the vesicle size was assumed.

### Cryo-TEM

Vesicle solution (5 μL droplet) was spread on a Lacey formar/carbon electron microscopy grid and preserved in a frozen-hydrated state by a rapid freezing in liquid ethane. The vitrification process was performed using FEI Vitrobot system with the setting of a single blot of 3 s, an offset of 1, and drain and waiting time of 1 s. Transmission electron microscopy (TEM) (JEOL 2100) with an accelerating voltage of 200 kV at magnifications in the range of × 10,000 to × 150,000 was used to image vesicles.

### Stability Studies

Physical stability studies of niosomes and liposomes, prepared with and without pHLIP, were carried out at two different storage temperatures (25 °C and 4 °C). The aim was to evaluate if significant changes in size and ζ-potential of the vesicles dispersion occur over a period of 90 days. Samples from each batch were withdrawn at definite time intervals (1, 30, 60, and 90 days) and the vesicles size and ζ-potential were determined as previously described.

Similarly, the vesicles stability was investigated also in the presence of biological fluids, such as human serum. The suspensions were contacted with 45% of human serum at 37 °C. At definite time intervals (0, 30 min, 1 h, 2 h, and 3 h), variations in vesicle size and ζ-potential were determined as described.

### Bilayer Characterization

Fluidity and microviscosity-polarity of niosomal and liposomal bilayers were accessed by fluorescence measurements of two fluorescent probes, diphenylhexatriene (DPH) and pyrene, respectively, located in the hydrophobic region of the bilayer membrane. Both DPH (2 mM) and pyrene (4 mM), 220 μL and 2.5 mg respectively, were added to the surfactant or phospholipid/cholesterol mixture before vesicle preparation [28]. Afterwards, pyrene-loaded vesicles were purified as previously described, while DPH-loaded vesicles were filtrated through progressively smaller pore size (from 5.0 to 0.22 μm).

The fluorescence anisotropy measurements were carried out at room temperature using an LS55 spectrofluorometer (PerkinElmer, MA, USA) at λ_exc_ = 400 nm and λ_em_ = 425 nm and the fluorescence anisotropy (*A*) of samples was calculated according to the following equation [29]:

$$ A=\frac{I_{vv}-{I}_{vh\kern0.5em }x\ G}{I_{vv}+2{I}_{vh}\ x\ G} $$

where *I*_*vv*_ and *I*_*vh*_ are the intensities of the emitted fluorescence (arbitrary units), respectively, parallel and perpendicular to the direction of the vertically polarized excitation light. The correction factor *G = I*_*hv*_*/I*_*hh*_ is the ratio between the vertically and the horizontally polarized emission components when the excitation light is horizontally polarized. The fluorescence anisotropy values are inversely proportional to membrane fluidity. Therefore, high fluorescence anisotropy value corresponds to a high structural order and/or low membrane fluidity [30].

In order to evaluate the microviscosity and the micropolarity of the vesicle bilayer, fluorescence experiments with pyrene were carried out with a Perkin-Elmer LS55 spectrofluorometer at λ_exc_ = 330 nm, recording the emission spectrum in the range 350 < λ_em_ < 550 nm [28]. The fine structure of pyrene fluorescence presents five peaks. The ratio between the intensities of the first (372 nm) and the third (382 nm) vibrational bands of the pyrene fluorescence spectrum, *I*_1_/*I*_3_, is related to the polarity of the pyrene environment [31]. Indeed, low values of the *I*_1_/*I*_3_ ratio correspond to a nonpolar environment. Further, pyrene molecules incorporated into the vesicle bilayer may form intramolecular excimers and this process is sensitive to the viscosity of the probe microenvironment [32]. Therefore, the excimer/monomer fluorescence intensity ratio, *I*_E_/*I*_M_, is related to microviscosity.

These studies were carried out on the vesicles prepared both with and without pHLIP and the obtained results were then compared.

### Calcein Release Studies

Non-ionic surfactant or phospholipid vesicles were loaded with calcein at concentration of 10^−2^ M. At this concentration, the calcein fluorescence is self-quenched [33]. Dequenching measurements were then used to determine the calcein release from the vesicle aqueous core. The fluorescent probe was loaded into the vesicles during hydration of the thin film by addition of 5 mL of the calcein aqueous solution. The vesicle suspensions were purified by gel permeation chromatography, contacted with 45% human serum or Hepes, and then loaded inside a cellulose membrane dialysis bag (cut-off molecular weight 8.000 Da from Spectra/Por®). The release experiments were carried out at 37 °C, under magnetic stirring in Hepes buffer (10 mM, pH 7.4) as an external medium. Aliquots of the external medium were withdrawn at different times over 0–24 h. From time to time, the withdrawn aliquots were reintroduced in the system [34].

The calcein release was monitored by measurements of the fluorescence of the medium using a Perkin-Elmer LS50B spectrofluorometer with λ_ex_ = 492 and λ_em_ = 520 nm. The reference value *F*_∞_ (arbitrary units), correlated to the total calcein amount entrapped in the vesicles [35], was determined after the disruption of the vesicles using isopropyl alcohol (1:1 *v*/*v*). The release percentages (*F*%) at each time point were obtained using the following equation:

$$ F\%={F}_t/{F}_{\infty }x\ 100 $$

where *F*_t_ (arbitrary units) is the fluorescence read at a specific time point *t*.

### Statistical Analysis

A one-way ANOVA was used for statistical analysis. A posteriori Bonferroni *t* test was carried out to evaluate the statistical significance of the ANOVA test. A *p* value < 0.05 was considered statistically significant. Results are the average of three experiments ± standard deviation.

## Results and Discussion

Tween20-, Span20-, and DMPC-vesicles, prepared with and without DSPE-pHLIP, were characterized for size and ζ-potential (Table 2).

According to the results presented in Table 2, the analyzed samples do not show significant variations in either size or ζ-potential, regardless the pHLIP presence.

**Fig. 1:**
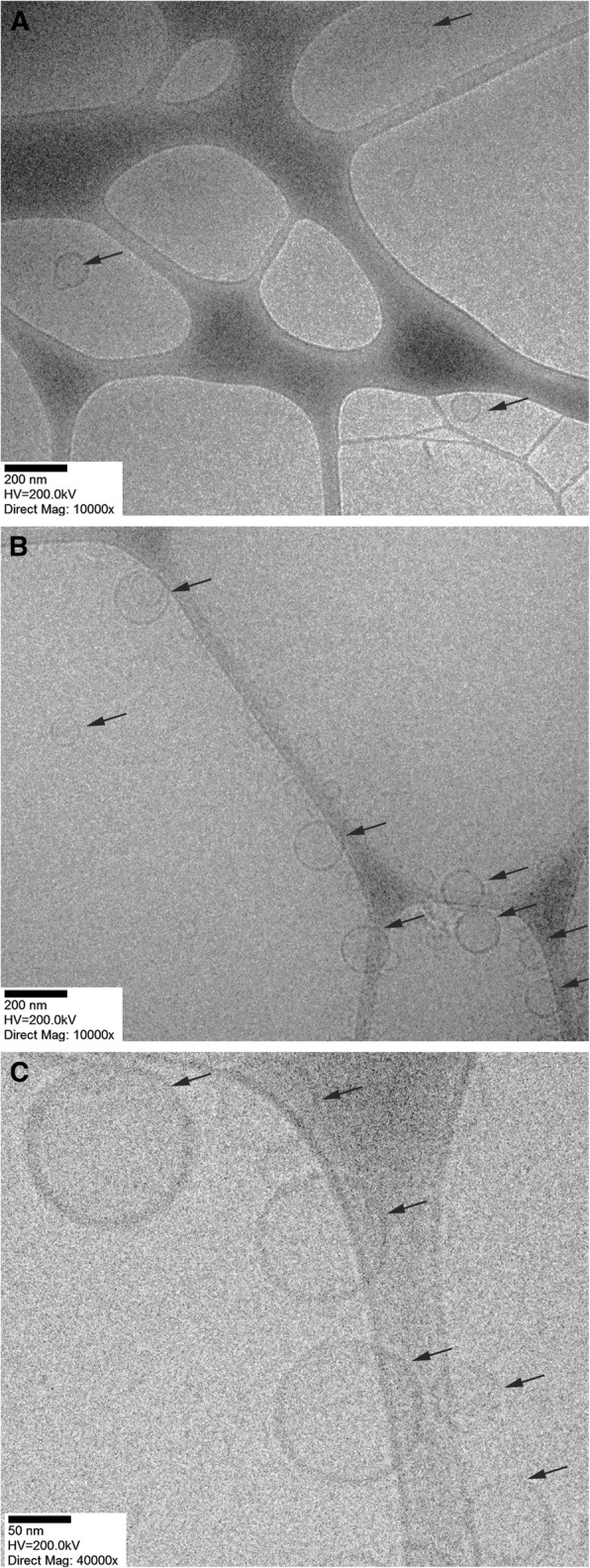
Representative cryo-TEM images of niosomes and liposomes coated with pHLIP. Images of NioSpan20-pHLIP niosomes (**a**) and LipoDMPC-pHLIP liposomes (**b**) obtained at × 10,000 magnification and LipoDMPC-pHLIP liposomes (**c**) obtained at × 40,000 magnification

The above reported size ranges were confirmed by means of a different analysis technique, such as the cryo-TEM (Fig. 1).

**Fig. 2:**
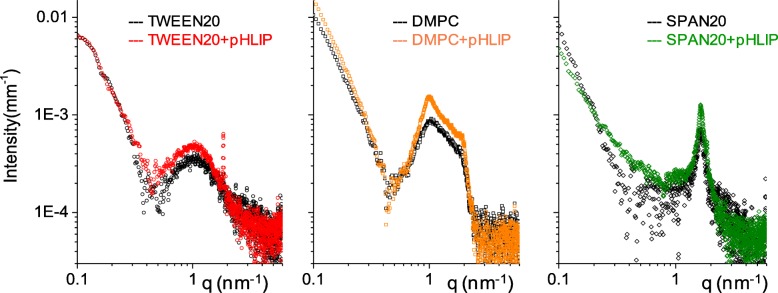
SAXS spectra. Intensity spectra of niosomes/vesicles without (black open symbols) and with DSPE-pHLIP (color symbols)

The internal structure of niosomes/vesicles with and without DSPE-pHLIP was determined by SAXS experiments. The SAXS intensity spectra, presented in Fig. 2, are different, indicating that each system has peculiar features.

Tween20-based niosomes are unilamellar, while DMPC vesicles display a certain degree of multilamellarity (as liposomes), as shown by the presence of the characteristic peak at *q* ≅ 1 nm^−1^, corresponding to an interlamellar distance of 6 nm (about 5 nm lipid bilayer and 1 nm water). Span20-based vesicles are definitely multilamellar, with a characteristic peak at *q* = 1.6 nm^−1^ corresponding to an interlamellar distance of 3.8 nm, matching twice the length of the Span20 molecule. Results indicate that the multilayered shell of Span20 vesicles is composed of adjacent bilayers with almost no interlamellar water.

The presence of DSPE-pHLIP (less than 1% mole fraction) does not dramatically affect the systems. The scattered intensity in all the investigated *q*-range is quite similar, indicating comparable fraction of structured material and comparable overall size of the vesicular particles.

DMPC-based vesicles show a slight increase in the number of bilayers within the liposome, as deducible by the intensity increase of the Bragg peak at *q* = 1 nm^−1^. The insertion of a low fraction of DSPE C18-chains, linked to each peptide molecule, does not change the thickness of the lipid bilayer, and mainly dictated by the large fraction of cholesterol with respect to DMPC (about 40–50%). At this concentration of cholesterol, lipid chains are organized in the liquid ordered (Lo) phase, and characterized by the decoupling of the weak lateral positional order of the chains with respect to the high orientational order along the chains and capable of hosting few C18-chains without any structural change [36]. Also Span20-based multilayered vesicles display no changes in their internal structure.

In the Tween20 + pHLIP system, a small amount of cholesterol crystallites is present, as shown by the typical sharp peak at *q* = 1.84 nm^−1^ (characteristic distance of 3.41 nm), and the vesicle structure is unaffected. The presence of few microcrystallites has been often found associated with Tween-based niosomes [37] in amount depending on composition, preparation procedure, and purification. In Fig. 2, the two experimental spectra of Tween20 and Tween20 + pHLIP vesicles are shown together with the fitting curves obtained by modeling the spherically closed bilayer with three concentric shells: the external headgroups, the chains, and the internal headgroups. The fitting average size of vesicles is about 168 nm in both systems, in agreement with DLS results. Structural parameters are the same for the two closed bilayers (see Additional file 1: Figure S1 and Table S2) except for the external shell: in the presence of DSPE-pHLIP, a slight decrease (5%) of the electron density of the headgroups is observed (from 0.42 to 0.40 e/Å^3^), together with an increase in the roughness of the layer. This result indicates that addition of DSPE-pHLIP mainly affects the external shell of the vesicles. This is consistent with a picture where the DSPE C18-chains insert into the bilayer and anchor the peptide to the vesicle surface, among the extended and ramified polyethylene-glycol headgroups. We note that the ability of pHLIP peptide itself (not linked to the lipid molecules) to interact with the external surface of vesicles at high pH and insert into bilayer at low pH has been recently observed by SAXS in the case of unsaturated phospholipid bilayers [38]. In the present study, the mole fraction of peptide is significantly lower and peptide is linked to lipids, as the goal is to design pHLIP-coated pH-sensitive drug nanovectors. The pHLIP association is driven by the hydrophobic interaction of the conjugated DSPE C18-chains inserting in the hydrophobic region of the nanovector. The pHLIP peptide is anchored and lies in the external headgroup region, prone to interact with target membranes at acidic pH and/or to release the nanovector content after bilayer rearrangement in low pH environment.

To investigate whether the pHLIP presence could affect bilayer microrheological properties (fluidity, microviscosity, and polarity), a lipophilic shell characterization was carried out. The characterization studies were conducted by using the fluorescent probe pyrene, added to the samples at the beginning of the preparation procedure. Due to its lipophilic nature, it inserts into the vesicle bilayer and provides information about the polarity and microviscosity of the membrane environment.

As shown in Table 3, in all of three samples sets, polarity values just slightly decrease in the presence of pHLIP compared to the counterpart vesicle without pHLIP, while microviscosity values show a threefold increase, for all vesicles.

**Table 3:** *I*_1_/*I*_3_ (polarity), I_E_/I_3_ (microviscosity) and fluorescence anisotropy values of the vesicle bilayer

Sample	Polarity (*I*_1_/*I*_3_)	Microviscosity (*I*_E_/*I*_3_)	Fluidity (anisotropy)
NioTween20	1.12	0.42	0.20
NioTween20-pHLIP	0.97	1.20	0.10
NioSpan20	0.97	0.38	0.30
NioSpan20-pHLIP	0.91	1.21	0.10
LipoDMPC	0.96	0.38	0.24
LipoDMPC-pHLIP	0.95	1.18	0.18

The bilayer was further characterized by measurements of fluorescence anisotropy of another lipophilic fluorescent probe, DPH, incorporated into lipids. These measurements reflect the probe movement and its orientation within the bilayer which gives information on the bilayer fluidity that can affect the vesicles capability to release their content [29]. Fluorescence anisotropy values are inversely proportional to the membrane fluidity. Hence, high fluorescence anisotropy value corresponds to a high structural order and/or low membrane fluidity, as in liquid-ordered phase [30].

**Fig. 3:**
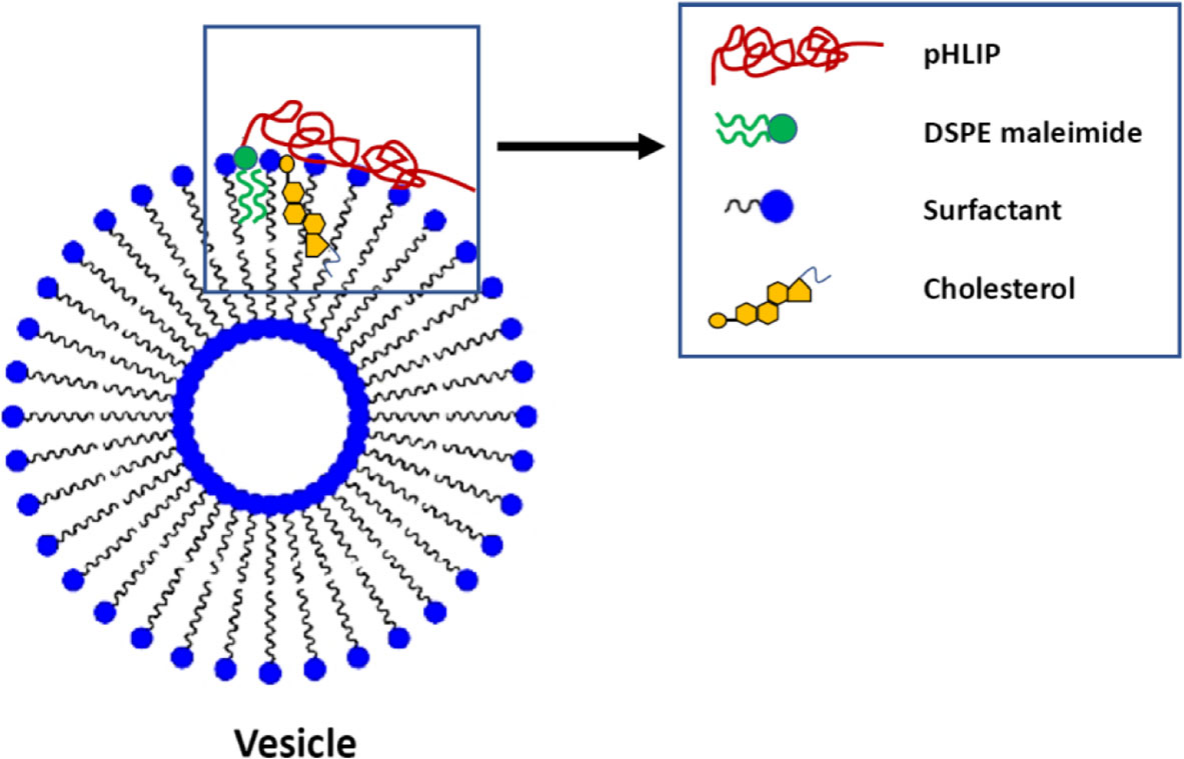
Representation of pHLIP interactions with vesicle membrane

The anisotropy values for each sample are shown in Table 3. The data indicate that the presence of the DSPE-pHLIP in the vesicles increases membrane fluidity, since the fluorescence anisotropy decreased. It is well known that the bilayer fluidity is affected not only by order and lateral organization of the apolar chains but also by the polar headgroups [31]. SAXS results on the three different systems show that DSPE-pHLIP molecules affect mainly the headgroup region, eventually causing a different packing of the polar heads. Structural results seem to match with microrheological properties of the bilayers in presence of associated DSPE-pHLIP as revealed by the insertion of two different probes. Results show an increased microviscosity as revealed by pyrene, a probe that could insert close to the outer region (pyrene log P is 4.88). On the other hand, DPH is assumed to be embedded in the core of the bilayers, oriented parallel to the lipid acyl chain axis or constrained in the center of the bilayer, parallel to the surface. It is largely sensitive to the angular reorientation of amphiphile acyl chains [39]. The increased fluidity, as revealed by DPH, could be related to both the effect of the insertion of DSPE chains among the other acyl chains and to the effect of pHLIP partition in the polar headgroups region, changing the packing properties of the surfactants. (Fig. 3).

Further characterization studies were conducted on the examined preparations. All the samples, prepared with and without pHLIP, were stored for 90 days at 4 °C temperature in order to evaluate stability of the vesicles over time, by measuring their size and ζ-potential variations (Additional file 1: Figure S2). No significant variations in size and ζ-potential were observed along the experiment; therefore, all the examined preparations resulted stable if stored at 4 °C temperature.

In addition, stability of the samples was examined in the presence of 90% (*v*/*v*) human serum and stored at 37 °C for 3 h (Additional file 1: Figure S3).

In contact with human serum, Tween20 vesicles showed an increase in size, although remained below 300 nm. The size and the PDI values were measured to be constant along the 3-h experiment, suggesting that the vesicles in suspension remained intact along the entire experiment.

Both the Span20 samples in contact with human serum showed an increase in size, especially in the preparation with pHLIP, while PDI values suggest a homogeneous size distribution (data not shown). This event can be due to the absorption of plasma proteins on the niosomal surface, as result of electrostatic interactions, however, without incurring in vesicles disruption. This hypothesis is supported by the ζ-potential decrease after the vesicles contact with human serum. This phenomenon occurs when the ζ-potential reaches slightly negative values, around the neutrality region, making the system to be unstable, and vesicles start aggregating.

As well as Tween20, DMPC vesicles show an increase in size in comparison to the pre-contact sample. However, this variation turned out to be non-significant and the size values maintained under 280 nm (DMPC) or under 240 nm (DMPC + pHLIP).

The release capability was evaluated for the samples prepared with and without pHLIP following the amount of calcein (hydrophilic probe) released from the vesicles over the time.

**Fig. 4:**
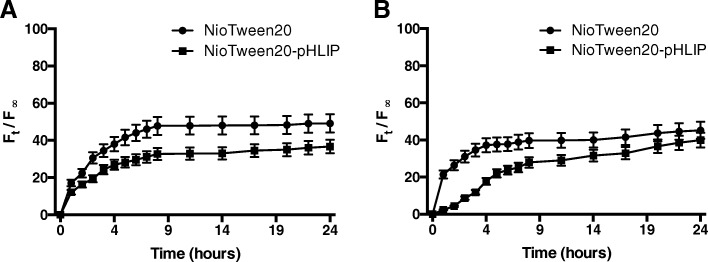
Calcein release profile. NioTween20 and NioTween20-pHLIP contacted with HEPES buffer (**a**) or human serum (**b**)

The studies were carried out at the temperature of 37 °C for a time period of 24 h, by exposing of the purified samples with either HEPES buffer or human serum. As shown in the Fig. 4, calcein release profiles turn out to be very similar for both Tween20 and Tween20-pHLIP in HEPES buffer or human serum.

The released amount of calcein was in between 30 and 50% indicating no differences in the release capabilities between the samples prepared with and without pHLIP.

Same results have been obtained for Span and DMPC samples (data not shown).

Comparable release profiles have been reported for different stimuli-responsive nanocarriers, such as thermo-sensitive cubosomes [40] or polymeric self-assembled nanocarriers (SANs) [41, 42].

In conclusion, the physico-chemical differences observed in terms of microviscosity and fluidity for the samples prepared with and without pHLIP (Table 3) do not affect their release capabilities (Fig. 4), according to the evidence that the insertion of DSPE-pHLIP molecules affect mainly the headgroup region. These data are in agreement with previously reported results showing that at neutral and high pHs, pHLIP is bound to the surface of liposomes made by 1-Palmitoyl-2-oleoyl-*sn*-glycero-3-phosphocholine (POPC) and does not induce fusion or membrane leakage.

## Conclusion

This study confirms the possibility of preparing pHLIP decorated vesicles. Samples are stable if stored at 4 °C temperature for at least 3 months and in the presence of serum. Furthermore, proposed nanovectors are able to entrap a hydrophilic probe and to modulate its release.

pHLIP vesicles were fully characterized in order to obtain fundamental understanding on nanocarrier features and facilitate the rational design of acidity sensitive nanovectors.

The pHLIP association is driven by the hydrophobic interaction of the conjugated DSPE C18-chains inserting in the hydrophobic region of the nanovector. The pHLIP peptide is anchored and lies in the external headgroup region, prone to interact with target membranes at acidic pH and/or to release the nanovector content after bilayer rearrangement in low pH environment.

According to these findings, proposed pHLIP decorated vesicles could be useful to obtain a prolonged (modified) release of biological active substances for targeting tumors and other acidic diseased tissues.

## Additional file

Additional file 1: **Table S1.** Parameters for the preparation of several samples with and without pHLIP. **Figure S1.** SAXS intensity spectra of TWEEN20 based vesicles without (black dots) and with (red dots) pHLIP, together with the fitting curves obtained with a multishell model. The core has the same electron density of the solvent (water) and the three shells are constituted by the inner headgroups layer, the hydrophobic layer, the outer headgroups layer. Parameters, thickness and electron density are reported in the Table S2. **Table S2.** Parameters, thickness and electron density obtained by SAXS analysis. **Figure S2.** Samples stability in terms of size and ζ-potential variations over time. **Figure S3.** Z-Average variations for all samples in contact with human serum. The ζ-potential values are around -7 mV for all samples. (DOCX 635 kb)

## Abbreviations

Chol: Cholesterol
Cryo-TEM: Cryo-transmission electron microscopy
DLS: Dynamic light scattering
DMPC: 1,2-Dimyristoyl-sn-glycero-3-phosphocholine
DPH: Diphenylhexatriene
pHLIP: pH (low) insertion peptide
SAXS: Small angle X-ray scattering
Span20: Sorbitan monolaurate
Tween20: Polyoxyethylenesorbitan monolaurate
