# Supplementary material for: Decoration of Nanovesicles with pH (Low) Insertion Peptide (pHLIP) for Targeted Delivery
Source: Nanoscale Res Lett. 2018 Dec 4;13:391. doi: 10.1186/s11671-018-2807-8 (PMC6279677; doi:10.1186/s11671-018-2807-8)
Supplement: Supplementary file 1 — Table S1. Parameters for the preparation of several samples with and without pHLIP. Figure S1. SAXS intensity spectra of TWEEN20 based vesicles without (black dots) and with (red dots) pHLIP, together with the fitting curves obtained with a multishell model. The core has the same electron density of the solvent (water) and the three shells are constituted by the inner headgroups layer, the hydrophobic layer, the outer headgroups layer. Parameters, thickness and electron density are reported in the Table S2. Table S2. Parameters, thickness and electron density obtained by SAXS analysis. Figure S2. Samples stability in terms of size and ζ-potential variations over time. Figure S3. Z-Average variations for all samples in contact with human serum. The ζ-potential values are around -7 mV for all samples. (DOCX 635 kb) [file 11671_2018_2807_MOESM1_ESM.docx]

**Additional file**

**Table S1.** Parameters for the preparation of several samples with and without pHLIP

| Sample | Rotavapor  Temperature (°C) | Sonicator | | |
| --- | --- | --- | --- | --- |
|  |  | Amplitude | Temperature (°C) | Time (min) |
| **NioTween20** | 25 | 16 | 60 | 5 |
| **NioSpan20** | 60 | 18 | 60 | 5 |
| **LipoDMPC** | 25 | 16 | 4 | 15 |

**Figure S1.** SAXS intensity spectra of TWEEN20 based vesicles without (black dots) and with (red dots) pHLIP, together with the fitting curves obtained with a multishell model.

The core has the same electron density of the solvent (water) and the three shells are constituted by the inner headgroups layer, the hydrophobic layer, the outer headgroups layer.

Parameters, thickness and electron density are reported in the Table S2.

**Table S2.** Parameters, thickness and electron density obtained by SAXS analysis

|  | Tween20 | Tween20 + pHLIP |
| --- | --- | --- |
| Core radius nm | 78 | 78 |
| Core electron density e/nm^3^ | 334 | 334 |
| Core polydispersity (Shultz) | 0.1 | 0.1 |
| Headgroups (inner layer) thickness nm | 1.4 | 1.4 |
| Headgroups (inner layer) electron density e/nm^3^ | 395 | 395 |
| Chains (hydrophobic layer) thickness nm | 3 | 3 |
| Chains (hydrophobic layer) electron density e/nm^3^ | 298 | 298 |
| Chains polydispersity | 0.1 | 0.1 |
| Headgroups (outer layer) thickness nm | 1.5 | 1.5 |
| Headgroups (outer layer) electron density e/nm^3^ | **420** | **400** |
| Headgroups (outer layer) polydispersity | **0** | **0.3** |

**Figure S2** Samples stability in terms of size and ζ-potential variations over time.

**Figure S3** Z-Average variations for all samples in contact with human serum.

The ζ-potential values are around -7 mV for all samples.
